# New species and suborder records reveal unusually high thaliacean (Chordata, Tunicata) diversity in the South China Sea

**DOI:** 10.3897/zookeys.1290.188516

**Published:** 2026-08-21

**Authors:** Yanjiao Lai, Zeqi Zheng, Mianrun Chen, Yehui Tan

**Affiliations:** 1 Nansha Islands Coral Reef Ecosystem National Observation and Research Station/South China Sea Development Research Institute, Ministry of Natural Resources (Remote Sensing Technology Application Center of South China Sea, MNR), Guangzhou 510300, China State Key Laboratory of Tropical Oceanography, Guangdong Provincial Key Laboratory of Applied Marine Biology, South China Sea Marine Biodiversity Collections, South China Sea Institute of Oceanology, Chinese Academy of Sciences Guangzhou China; 2 State Key Laboratory of Tropical Oceanography, Guangdong Provincial Key Laboratory of Applied Marine Biology, South China Sea Marine Biodiversity Collections, South China Sea Institute of Oceanology, Chinese Academy of Sciences, Guangzhou 510301, China Department of Oceanography & Coastal Sciences, College of the Coast & Environment, Louisiana State University Baton Rouge United States of America; 3 Department of Oceanography & Coastal Sciences, College of the Coast & Environment, Louisiana State University, Baton Rouge, LA, USA University of Chinese Academy of Sciences Beijing China; 4 University of Chinese Academy of Sciences, Beijing, 100049, China Nansha Islands Coral Reef Ecosystem National Observation and Research Station/South China Sea Development Research Institute, Ministry of Natural Resources (Remote Sensing Technology Application Center of South China Sea, MNR) Guangzhou China

**Keywords:** Doliolids, gelatinous zooplankton, marine biodiversity, salps, taxonomy

## Abstract

In the context of global biodiversity decline, the monitoring and assessment of marine biodiversity have gained increasing importance. Thaliacea, a group of filter-feeding gelatinous zooplankton, play crucial roles in the carbon cycle and pelagic food webs, yet their diversity remains poorly documented due to challenges in sampling and identification. This knowledge gap is particularly evident in the South China Sea, a highly dynamic subtropical marginal sea. To achieve a comprehensive assessment of thaliacean diversity in the South China Sea, we conducted two cruises in the western South China Sea during the summers of 2020 and 2022. A total of 28 thaliacean species were recorded, representing the highest species richness ever reported from a single study in the South China Sea. Among these, five species are newly recorded, all belonging to the family Doliolidae (order Doliolida): *Dolioletta mirabilis* Korotneff, 1891, *Dolioletta tritonis* Herdman, 1888, Doliolina (Doliolina) krohni Herdman, 1888, Doliolina (Doliolina) sigmoides Garstang, 1933, and Doliolina (Doliolinetta) indica Neumann, 1906. In addition, taxa of the suborder Doliopsidina are reported in the South China Sea for the first time. This study expands current knowledge of Thaliacea diversity and distribution in the South China Sea, highlighting the need to reassess their diversity and distribution using integrated methodologies.

## Introduction

Knowledge of biodiversity provides a critical foundation for understanding ecosystem structures and functions (Duffy and Stachowicz 2006; Da Silva et al. 2025). The sharp global biodiversity decline driven by climate change and human disturbances creates an urgent need for comprehensive biodiversity monitoring (Ratnarajah et al. 2023; Keck et al. 2025). However, this poses a particular challenge for gelatinous zooplankton due to their fragile and easily damaged bodies, which impedes a comprehensive understanding of their diversity and ecological functions (Biard et al. 2016; Stukel et al. 2018; Greer and Chiaverano 2026).

Thaliaceans are an important group of filter-feeding gelatinous zooplankton containing three orders: Pyrosomatida, Salpida, and Doliolida, with 78 known valid species (WoRMS 2026). Their remarkable reproductive capacity and short generation times facilitate the frequent formation of dense swarms. In recent years, the crucial role of thaliaceans in the marine biological carbon pump and ecosystem has garnered significant scientific interest. They efficiently transfer carbon to the deep sea through the production of substantial quantities of fast-sinking fecal pellets and carcasses (Stenvers et al. 2021; Décima et al. 2023; Orlov and Pakhomov 2024). Their dense aggregations can rapidly consume primary productivity, thereby accelerating the transfer of energy to higher trophic levels (Batta-Lona et al. 2024). Moreover, thaliaceans can have cascading effects on pelagic food web structure by competing for phytoplankton and preying on other zooplankton eggs and larvae (Köster and Paffenhöfer 2022; Pietzsch et al. 2023).

Despite this growing recognition of their ecological importance, studies on the diversity of thaliaceans remain scarce. This is particularly true for species within the order Doliolida. For instance, although 25 species have been documented (WoRMS 2026), only a few, such as *Doliolum denticulatum*, *Doliolum nationalis*, and *Dolioletta gegenbauri*, have received widespread attention. This gap is largely attributed to the fact that these gelatinous organisms are highly susceptible to damage during both capture and subsequent preservation processes, posing significant challenges to accurate species identification.

The South China Sea (SCS), the largest subtropical marginal sea, is influenced by a complex interplay of dynamic forces, including currents (e.g., the Kuroshio and China Coastal Currents) and other physical processes such as fronts, upwelling, and eddies (Chu and Wang 2003; Su 2004). Combined with the seasonal monsoon effects, the SCS harbors diverse and unique ecological niches, resulting in a high diversity of plankton (Guo et al. 2025; Liu et al. 2025; Tao et al. 2025). These characteristics establish the SCS as a key region for understanding plankton diversity in marginal seas. However, research on thaliaceans in the SCS remains scarce, and detailed distribution and associated environmental information are available for only a few species (e.g., *D. denticulatum* and *D. gegenbauri* within the Doliolida; *Thalia democratica* and *Iasis cylindrica* within the Salpida), while the majority of other recorded species, particularly within Doliolida (e.g., *Doliolina obscura*), lack detailed data. Furthermore, the few existing studies were conducted more than a decade ago (Table 1). Given the ongoing environmental and plankton community shifts under global climate change (Cai et al. 2025; Wu et al. 2025), it remains unknown whether the thaliacean fauna of the SCS has undergone significant alterations in composition or biogeography. This gap hinders a comprehensive assessment of their distribution and ecological role, as well as the identification of local extinctions, non-native species, and biogeographic shifts.

**Table 1.** Summary of primary historical studies on Thaliacea in the South China Sea.

**Sample Time**	**Region**	**Net length (cm)**	**Mouth diameter (cm)**	**Mesh size (μm)**	**Total species number**	**Reference**
Sep. 1983; Apr., Aug., and Dec. 1984	central SCS (12–20°N, 111–118°E)	270	80	500	23	Lin and Lin 2006
Mar., May, Jul., Oct., and Dec. 1997	coastal waters southwest of Taiwan (ca 22°10'N, 120°10'E)		56	330	17	Tew and Lo 2005
Dec. 1997; May 1998; Jun.–Jul. 1999	eastern waters of the Taiwan Strait (23°30'N–25°30'N, 118°30'E–121°00'E)	270	80	505	11	Zhang et al. 2003b
Feb., Sep., Nov., and Dec. 1998; Apr.–May 1999	Beibu Gulf (16.5°N–21.5°N, 106°E–110°E)	280	80	505	9	Wang et al. 2010
Nov. and Dec. 2001	Nanwan Bay, Taiwan (21°52'12"N–21°57'7"N, 120°43'12"E–120°51'0"E)	180	45	200 and 333	16	Zhang et al. 2003a
Feb. and Aug. 2004	waters around Taiwan (21°N–26°N, 118°E–123°E)		160	330	18	Liao et al. 2013
Jul.–Aug. 2006; Dec. 2006 – Jan. 2007	northern SCS (17°N–22°N, 109°E–114°E)		80	505	14	Li et al. 2011
May–Jun 2009; Nov 2010; Aug–Sep 2012	central SCS (6°N–18°N, 108°E–119°E)	280	80	505	13	Lai et al. 2024
Aug. and Nov. 2014; Mar. and May 2015	waters adjacent to the first and second nuclear power plants in Taiwan (25.2°N–25.34°N, 121.58°E–121.72°E)	180	45	333	4	Franco et al. 2016
Jul. 2015	waters surrounding Taiwan	180	45	333	13	Franco et al. 2017
Mar., Jul.–Aug., and Nov. 2014; Jan. 2015	sea around Zhongsha and Xisha Islands in the SCS (12°30'N–17°32'N, 110°00'E–110°11'E)	280	80	505	18	Li et al. 2016

To address this knowledge gap, the present study aimed to systematically assess the thaliacean community in the SCS via two summer cruises in 2020 and 2022. This study provides fundamental information on thaliacean diversity changes in the SCS under climate change, offering a scientific basis for future ecological monitoring and conservation strategies.

## Materials and methods

**Figure 1.**
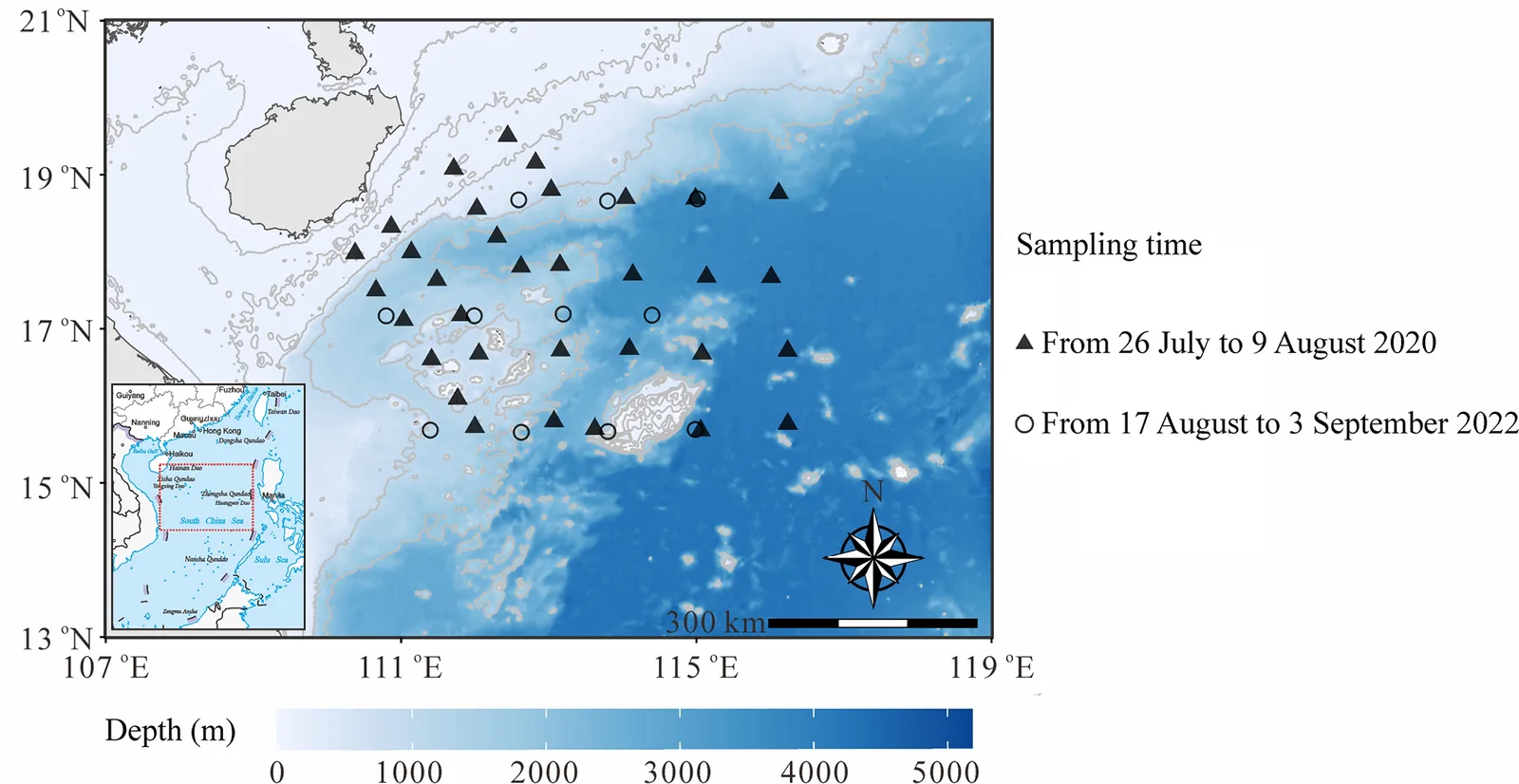
Sampling stations in the western South China Sea from 26 July to 9 August 2020, and from 17 August to 3 September 2022.

Thaliacea samples were collected during two cruises aboard the research vessel *Shiyan 3* in the western SCS (15°N–20°N, 110°E–117°E) from 26 July to 9 August 2020, and from 17 August to 3 September 2022 (Fig. 1). Vertical trawling for zooplankton was performed at a depth of 200 m, or from the bottom to the surface when the water depth was less than 200 m, using a plankton net with an 80 cm mouth diameter and a mesh size of 330 μm, equipped with a flowmeter (Digital Flow Meter 438115, HYDRO-BIOS, Germany). The net towing speed was maintained at 0.8 m s^-1^. The samples were preserved with 5% formaldehyde solution. Thaliacean specimens were sorted, identified, and quantified under a stereo microscope (Leica M165C, Leica Microsystems, Germany), following established references (Godeaux 1998a, 1998b, 2003; Adam and Ishak 2018; Ishak et al. 2018). To facilitate identification and quantification, some samples were stained with ~ 0.02% Rose Bengal sodium salt for 12 h. Thaliacea abundance was expressed as the number of individuals per cubic meter (ind m^-3^). The occurrence rate (%) of each species was defined as the proportion of samples where the species occurred to the total number of samples.

We obtained global records of Thaliacea species from several key biodiversity databases, including the Global Biodiversity Information Facility (https://www.gbif.org/), Ocean Biodiversity Information System (https://obis.org/), Jellyfish Database Initiative (https://doi.org/10.1575/1912/7191), SeaLifeBase (https://www.sealifebase.ca/), and World Register of Marine Species (https://www.marinespecies.org/).

## Results

### Environmental background

Remote sensing maps and in situ measurements indicated complex hydrological dynamics in the SCS during the sampling periods, resulting in diverse environmental conditions (Suppl. material 1). During the summer 2020 sampling period, environmental conditions were influenced by a low-salinity plume from the Mekong River in the southwest and a warm eddy extending across the region. Sea surface temperature ranged from 29.97–31.65 °C, with cooler waters in the west associated with the Qiongdong Upwelling and warmer waters in the east within the eddy center. Sea surface salinity varied between 33.72–34.23 ppt, showing lower values in the south consistent with the river plume. Sea surface chlorophyll *a* concentrations (0.03–0.40 μg L^-1^) were higher in the western upwelling area and lower in the eastern eddy-influenced region. In 2022, sea surface temperature ranged from 29.07–30.33 °C, sea surface salinity was lower in the south (33.19–33.52 ppt), and sea surface chlorophyll *a* concentrations (0.07–0.10 μg L^-1^) decreased from west to east, with the southern stations located at the edge of a warm eddy.

### Thaliacean species

A total of 4090 individuals were identified. Specimens of the order Pyrosomatida were not identified to species level, with occurrence rates of 12.12% in 2020 and 27.27% in 2022 (Table 2). Within the order Salpida, all individuals except those with damaged morphology were identified to the species level. The 16 identified species belong to eight genera in the family Salpidae (Table 2). In 2020, *Thalia orientalis* exhibited the highest occurrence rate of 96.97%, followed by *Brooksia rostrata* (42.42%) and *T. democratica* (39.39%). In 2022, *T. orientalis* again showed the highest occurrence rate (100%), followed by *T. democratica* (81.82%) and *B. rostrata* (63.64%).

**Table 2.** Occurrence rate and mean abundance of thaliaceans identified in this study.

**Order**	**Genus**	**Species**	**2020**		**2022**	
			**Occurrence rate (%)**	**Average abundance (ind m^-3^)**	**Occurrence rate (%)**	**Average abundance (ind m^-3^)**
Doliolida	Dolioletta	*D. gegenbauri*	36.36	0.03 ± 0.08	90.91	0.16 ± 0.20
Doliolida	Dolioletta	*D. mirabilis*	3.03	0.01 ± 0.05	0	0 ± 0
Doliolida	Dolioletta	*D. tritonis*	3.03	0.01 ± 0.05	0	0 ± 0
Doliolida	Dolioletta	*Dolioletta* spp.	78.79	0.15 ± 0.14	90.91	0.12 ± 0.09
Doliolida	Doliolina	*D. indica*	57.58	0.04 ± 0.05	36.36	0.03 ± 0.06
Doliolida	Doliolina	*D. krohni*	72.73	0.09 ± 0.09	63.64	0.12 ± 0.19
Doliolida	Doliolina	*D. muelleri*	0	0 ± 0	9.09	0 ± 0.01
Doliolida	Doliolina	*D. obscura*	63.64	0.05 ± 0.07	81.82	0.1 ± 0.14
Doliolida	Doliolina	*D. separata*	9.09	0 ± 0.01	45.45	0.05 ± 0.07
Doliolida	Doliolina	*D. sigmoides*	6.06	0.01 ± 0.03	27.27	0.02 ± 0.06
Doliolida	Doliolina	*Doliolina* spp.	100	0.35 ± 0.19	100	0.57 ± 0.55
Doliolida	Dolioloides	*D. rarum*	12.12	0.01 ± 0.01	81.82	0.25 ± 0.28
Doliolida	Dolioloides	*Dolioloides* spp.	54.55	0.06 ± 0.09	90.91	0.15 ± 0.21
Doliolida	Doliolum	*D. denticulatum*	100	0.32 ± 0.39	100	0.22 ± 0.19
Doliolida	Doliolum	*D. nationalis*	15.15	0.05 ± 0.19	27.27	0.01 ± 0.02
Doliolida	Doliolum	*Doliolum* spp.	81.82	0.09 ± 0.10	90.91	0.1 ± 0.08
Doliolida	Undefined	Doliopsidina spp.	48.48	0.08 ± 0.15	54.55	0.11 ± 0.15
Salpida	Brooksia	*B. rostrata*	42.42	0.04 ± 0.07	63.64	0.2 ± 0.27
Salpida	Cyclosalpa	*C. bakeri*	3.03	0 ± 0.02	0	0 ± 0
Salpida	Cyclosalpa	*C. floridana*	6.06	0 ± 0.01	9.09	0.01 ± 0.02
Salpida	Cyclosalpa	*C. pinnata*	3.03	0 ± 0.02	9.09	0 ± 0.01
Salpida	Cyclosalpa	*C. polae*	9.09	0.01 ± 0.06	0	0 ± 0
Salpida	Cyclosalpa	*C. virgula*	3.03	0 ± 0.010	0	0 ± 0
Salpida	Iasis	*I. cylindrica*	12.12	0.02 ± 0.08	18.18	0.05 ± 0.13
Salpida	Pegea	*P. confoederata*	0	0 ± 0	9.09	0.01 ± 0.04
Salpida	Ritteriella	*R. amboinensis*	6.06	0 ± 0.01	0	0 ± 0
Salpida	Salpa	*S. fusiformis*	6.06	0.01 ± 0.07	0	0 ± 0
Salpida	Thalia	*T. cicar*	6.06	0 ± 0.01	18.18	0.01 ± 0.02
Salpida	Thalia	*T. democratica*	39.39	0.03 ± 0.05	81.82	0.22 ± 0.35
Salpida	Thalia	*T. orientalis*	96.97	0.83 ± 0.72	100	2.68 ± 2.49
Salpida	Thalia	*T. rhomboides*	36.36	0.06 ± 0.18	36.36	0.16 ± 0.38
Salpida	Thalia	*T. sibogae*	6.06	0 ± 0.01	27.27	0.02 ± 0.04
Salpida	Thalia	*Thalia* spp.	57.58	0.06 ± 0.10	100	0.21 ± 0.24
Salpida	Traustedtia	*T. multitentaculata*	12.12	0.04 ± 0.14	9.09	0 ± 0.01
Pyrosomatida	Undefined	Pyrosomatida spp.	12.12	0.01 ± 0.02	27.27	0.17 ± 0.38
Undefined	Undefined	Undefined	57.58	0.07 ± 0.10	63.64	0.06 ± 0.07

For the order Doliolida, individuals belonging to the suborder Doliopsidina were not identified to species, with occurrence rates of 48.48% in 2020 and 54.55% in 2022 (Table 2). In the suborder Doliolidina, specimens in the phorozooid and gonozooid stages were identified to species, whereas those in other stages or with damaged morphology were identified only to genus. A total of 12 species were identified, representing four genera (Table 2). In 2020, *D. denticulatum* had the highest occurrence rate (100%), followed by Doliolina (Doliolina) krohni (72.73%) and *D. obscura* (63.64%). In 2022, *D. denticulatum* again recorded the highest occurrence rate (100%), followed by *D. gegenbauri* (90.91%), *Dolioloides rarum* (81.82%), and *D. obscura* (81.82%).

**Figure 2.**
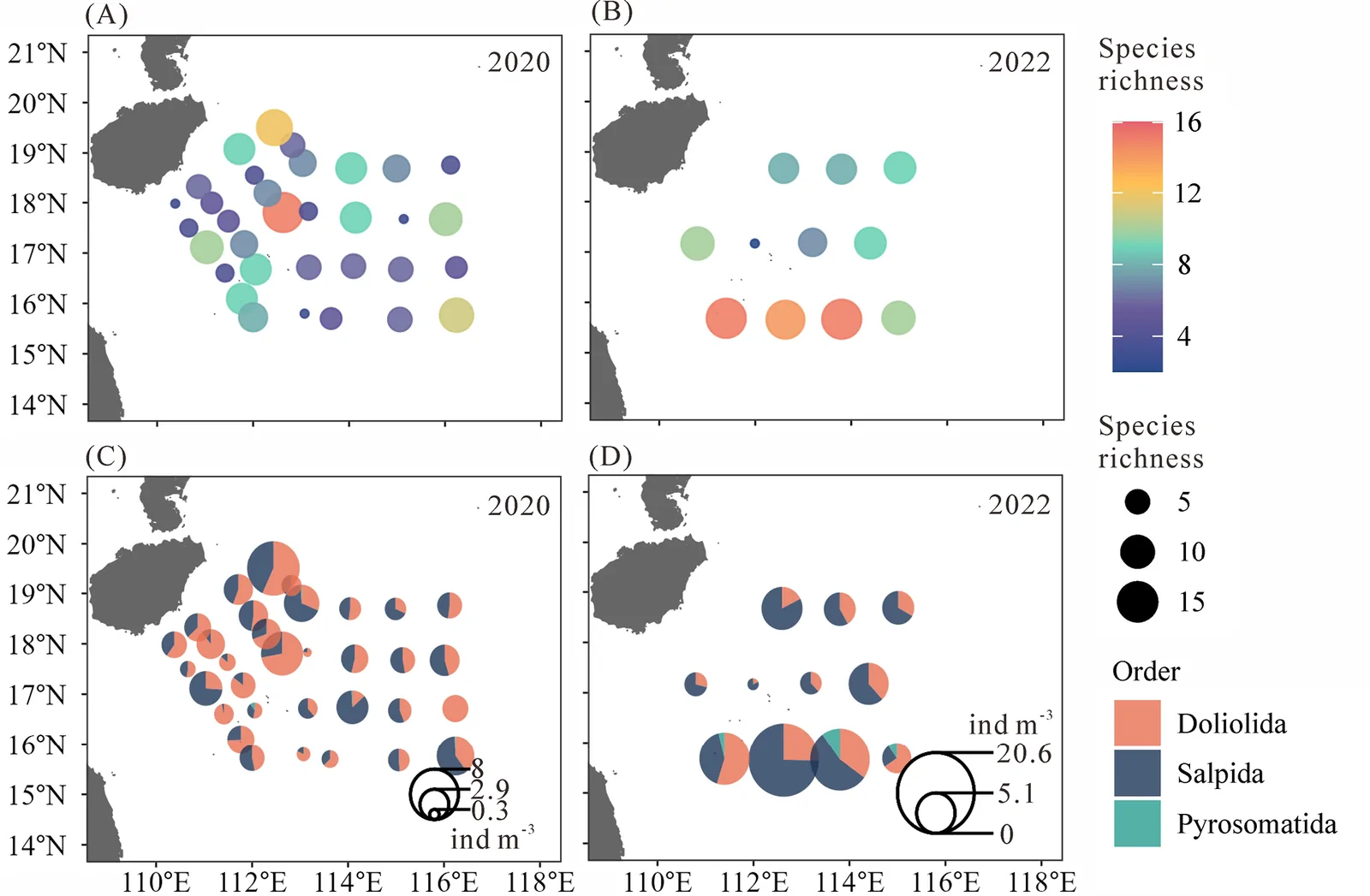
Spatial distribution of thaliacean species richness in 2020 and 2022 and total thaliacean abundance in 2020 and 2022 during the sampling period. **A**. Species richness in 2020; **B**. Species richness in 2022; **C**. Total abundance in 2020; **D**. Total abundance in 2022.

In 2020, species richness ranged from 3–15, showing considerable regional variation. The highest value was recorded at station S10. Stations in the northwest and southwest exhibited relatively high species numbers (>8), while richness in the southeastern area was generally below 7 (Fig. 2A). In 2022, species richness varied between 2–15. Stations along the edge of the warm eddy showed higher species numbers (≥14), while other regions exhibited less variation, with values of ~ 8 (Fig. 2B).

Total thaliacean abundance in 2020 was generally higher at southwestern stations, peaking at 9.63 ind m^-3^, whereas southern stations had lower abundances, mostly below 3 ind m^-3^ (Fig. 2C). In 2022, high total thaliacean abundance was observed along the edge of the warm eddy, reaching a maximum of 17.22 ind m^-3^, while stations on the central transect showed lower abundances, with a minimum of 0.45 ind m^-3^ (Fig. 2D).

### Morphology and distribution of newly recorded species

Compared to historical records in the SCS (Table 1), this study reports the highest species richness of Thaliacea documented in the region to date. A total of five newly recorded species were identified, all belonging to the family Doliolidae within the order Doliolida: *Dolioletta mirabilis* and *Dolioletta tritonis* in the genus *Dolioletta*, and *D. krohni*, Doliolina (Doliolina) sigmoides, and Doliolina (Doliolinetta) indica in the genus *Doliolina*. In addition, taxa of the suborder Doliopsidina are reported in the SCS for the first time.

### Taxonomic account

**Phylum: Chordata Haeckel, 1874**

**Subphylum: Tunicata Lamarck, 1816**

**Class: Thaliacea Van der Hoeven, 1850**

**Order: Doliolida Delage & Hérouard, 1898**

**Suborder: Doliolidina Godeaux, 1996**

**Family: Doliolidae Bronn, 1862**

#### Genus: *Dolioletta* Garstang, 1933

##### Dolioletta mirabilis

Taxon classificationAnimaliaDoliolidaDoliolidae

Korotneff, 1891

639DD3F1-1DC3-5D9D-8DBC-9F1938E5D3F8

Fig. 3

###### Material examined.

China • 6 gonozooids; South China Sea; 17.806°N, 112.625°E; 28 Jul. 2020; Y.J. Lai leg.; SDRI 202001, 202003, 202009, 202012, 202019, 202020 • 2 phorozooids; same data as for preceding; SDRI 202007, 202013.

###### Description.

**Figure 3.**
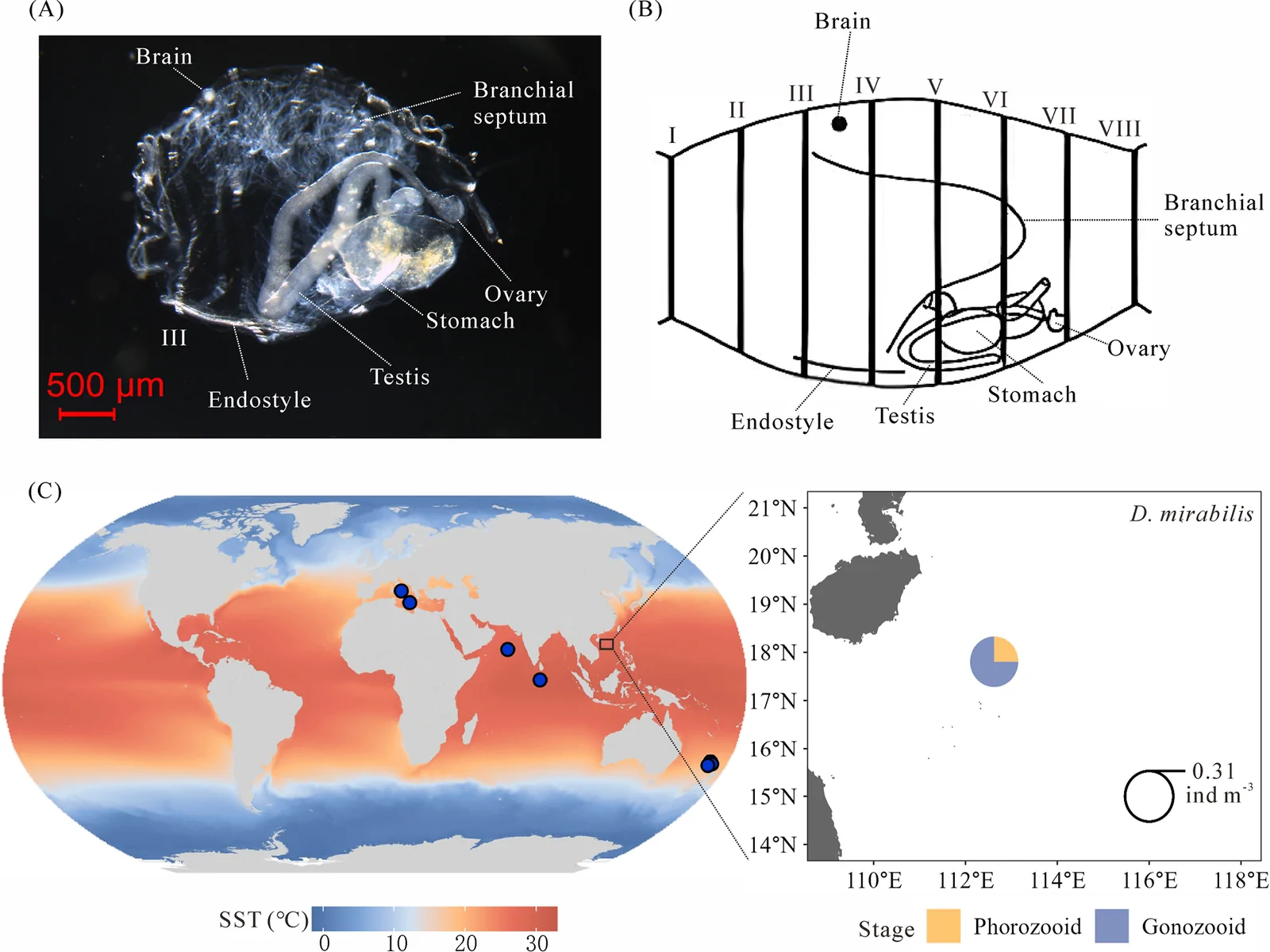
Gonozooid of *Dolioletta mirabilis* and its distribution. **A**. Light micrograph of the specimen; **B**. Schematic diagram adapted from Godeaux (1998a); **C**. Distribution of *D. mirabilis* in the South China Sea based on the present study and in the global ocean compiled from historical literature and biodiversity databases, including the Global Biodiversity Information Facility (GBIF), Ocean Biodiversity Information System (OBIS), Jellyfish Database Initiative (JeDI), SeaLifeBase, and WoRMS. Dots on the global map represent approximate occurrence locations.

Length 1.1–3.3 mm. Short endostyle extending from MII 4/5 to MIV 1/2. Branchial septum starting from MIII, ventrally close to MVI, and ending close to MIV. Brain located between MIII and MIV. Tubular testis coiled on the left side of the stomach; ovary located anterior to MVII. MVI thin or ventrally open (Godeaux 2003). Intestine dextrally coiled (Fig. 3A, B).

###### Remarks.

This species is primarily distinguished from the commonly occurring *D. gegenbauri* by the position and morphology of the tubular testis, which extends straight in *D. gegenbauri* but is coiled near the stomach in *D. mirabilis*.

###### Distribution.

In this study, this species was only found in S10 (17.806°N, 112.625°E) during the summer of 2020, with abundance of 0.31 ind m^-3^ (Fig. 3C). According to the historical literature and biodiversity databases, this species is found in the Mediterranean, Indian Ocean, New Zealand (Godeaux 2003), Naples, tropical Atlantic Ocean, and the south of the Arabian Sea (Purushothaman et al. 2018).

##### Dolioletta tritonis

Taxon classificationAnimaliaDoliolidaDoliolidae

Herdman, 1888

8E1A1911-6222-531D-828A-C347F81E69AD

Fig. 4

###### Material examined.

China • 10 gonozooids; South China Sea; 17.806°N, 112.625°E; 28 Jul. 2020; Y.J. Lai leg.; SDRI 202001, 202003, 202005, 202006, 202008, 202009, 202010, 202012, 202019, 202020 • 5 phorozooids; same data as for preceding; SDRI 202004, 202007, 202013, 202014, 202015.

###### Description.

Length 1.1–3.9 mm. Endostyle extending from MII 1/2 to MIV 1/2. Branchial septum starting from MII to MVI and ending at MIV 1/2. Brain located between MIII and MIV. Testis tubular, running obliquely and starting from MII. Intestine dextrally coiled.

###### Remarks.

**Figure 4.**
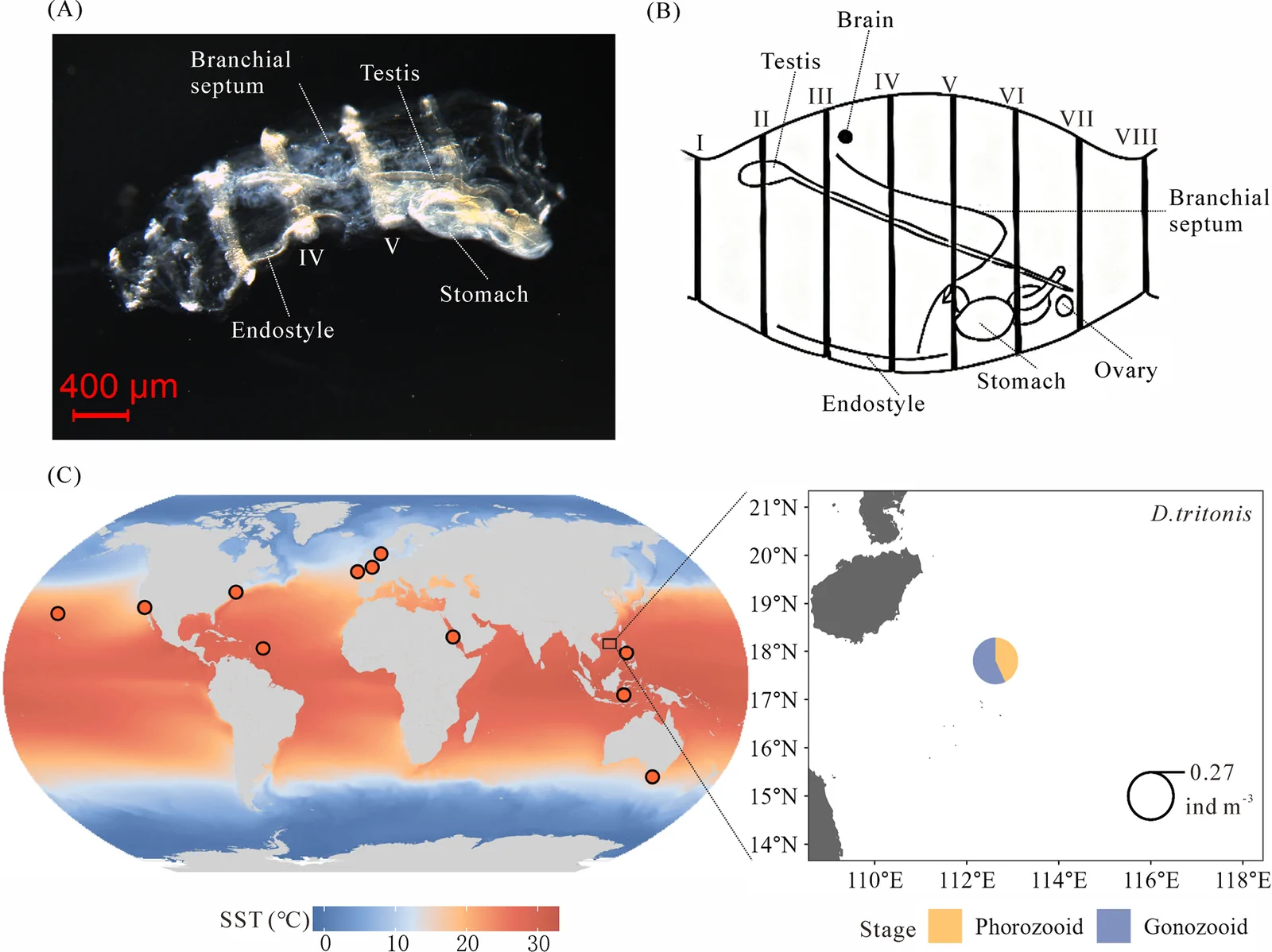
Gonozooid of *Dolioletta tritonis* and its distribution. **A**. Light micrograph of the specimen; **B**. Schematic diagram adapted from Godeaux (1998a); **C**. Distribution of *D. tritonis* in the South China Sea based on the present study and in the global ocean compiled from historical literature and biodiversity databases, including the GBIF, OBIS, JeDI, SeaLifeBase, and WoRMS. Dots on the global map represent approximate occurrence locations.

The position of branchial septum differs from that reported in previous studies. In the present study, the branchial septum extends from MII to MVI (Fig. 4A), in contrast to earlier descriptions in which it extended from MIII dorsally to MIV 1/2 ventrally (Fig. 4B; Godeaux 1998a, 2003; Purushothaman et al. 2018). This species closely resembles *D. gegenbauri*, except that the branchial septum is ventrally attached between MIV and MV.

###### Distribution.

In this study, this species was only found in S10 (17.806°N, 112.625°E) during the summer of 2020, with an abundance of 0.27 ind m^-3^ (Fig. 4C). According to the historical literature and biodiversity databases, this species was found in the Bass Strait, Indian Ocean, Pacific Ocean, North Atlantic (Purushothaman et al. 2018), the Gulf of Aqaba (Godeaux 2003), the English Channel, North Sea (Harant and Vernières 1938), Red Sea (Godeaux 1987), and the North Pacific (Tokioka 1960).

#### Genus: *Doliolina* Borgert, 1894

**Subgenus: Doliolina (Doliolina) Garstang, 1933**

##### Doliolina (Doliolina) krohni

Taxon classificationAnimaliaDoliolidaDoliolidae

Herdman, 1888

5AA97142-A527-57DF-8E5C-5377AE29DA07

Fig. 5

###### Material examined.

China • 3 gonozooids; South China Sea; 18.697°N, 114.044°E; 26 Jul. 2020; Y.J. Lai leg.; SDRI 202136, 202137, 202138 • 1 phorozooid; same data as for preceding; SDRI 202134 • 2 gonozooids; South China Sea; 19.078°N, 111.714°E; 27 Jul. 2020; Y.J. Lai leg.; SDRI 202174, 202175 • 2 phorozooids; same data as for preceding; SDRI 202173, 202176 • 2 gonozooids; South China Sea; 18.558°N, 112.028°E; 27 Jul. 2020; Y.J. Lai leg.; SDRI 202177, 202182 • 2 gonozooids; South China Sea; 19.503°N, 112.444°E; 27 Jul. 2020; Y.J. Lai leg.; SDRI 202163, 202167 • 1 phorozooid; same data as for preceding; SDRI 202169 • 1 gonozooid; South China Sea; 18.194°N, 112.300°E; 28 Jul. 2020; Y.J. Lai leg.; SDRI 202191 • 13 gonozooids; South China Sea; 17.806°N, 112.625°E; 28 Jul. 2020; Y.J. Lai leg.; SDRI 202001, 202003, 202005, 202006, 202008, 202009, 202010, 202012, 202017, 202018, 202019, 202020, 202024 • 9 phorozooids; same data as for preceding; SDRI 202004, 202007, 202011, 202013, 202014, 202015, 202021, 202022, 202023 • 5 gonozooids; South China Sea; 17.703°N, 114.139°E; 28 Jul. 2020; Y.J. Lai leg.; SDRI 202030, 202031, 202032, 202033, 202034 • 1 phorozooid; same data as for preceding; SDRI 202026 • 2 gonozooids; South China Sea; 16.675°N, 115.078°E; 29 Jul. 2020; Y.J. Lai leg.; SDRI 202052, 202053 • 1 gonozooid; South China Sea; 17.672°N, 116.014°E; 29 Jul. 2020; Y.J. Lai leg.; SDRI 202036 • 1 phorozooid; same data as for preceding; SDRI 202039 • 3 gonozooids; South China Sea; 16.714°N, 116.236°E; 29 Jul. 2020; Y.J. Lai leg.; SDRI 202042, 202043, 202044 • 1 phorozooid; same data as for preceding; SDRI 202048 • 6 gonozooids; South China Sea; 16.675°N, 112.056°E; 30 Jul. 2020; Y.J. Lai leg.; SDRI 202058, 202061, 202062, 202066, 202067, 202073 • 1 phorozooid; same data as for preceding; SDRI 202068 • 1 gonozooid; South China Sea; 16.717°N, 113.161°E; 30 Jul. 2020; Y.J. Lai leg.; SDRI 202057 • 1 phorozooid; same data as for preceding; SDRI 202056 • 4 gonozooids; South China Sea; 18.325°N, 110.867°E; 4 Aug. 2020; Y.J. Lai leg.; SDRI 202094, 202095, 202096, 202097 • 1 phorozooid; South China Sea; 17.994°N, 111.139°E; 4 Aug. 2020; Y.J. Lai leg.; SDRI 202090 • 2 gonozooids; South China Sea; 17.633°N, 111.486°E; 5 Aug. 2020; Y.J. Lai leg.; SDRI 202088, 202089 • 2 gonozooids; South China Sea; 17.175°N, 111.814°E; 5 Aug. 2020; Y.J. Lai leg.; SDRI 202076, 202077 • 2 phorozooids; same data as for preceding; SDRI 202074, 202086 • 1 gonozooid; South China Sea; 15.681°N, 115.058°E; 6 Aug. 2020; Y.J. Lai leg.; SDRI 202142 • 2 phorozooids; South China Sea; 15.767°N, 116.239°E; 6 Aug. 2020; Y.J. Lai leg.; SDRI 202153, 202159 • 1 gonozooid; South China Sea; 15.700°N, 113.625°E; 7 Aug. 2020; Y.J. Lai leg.; SDRI 202139 • 1 gonozooid; South China Sea; 16.603°N, 111.414°E; 8 Aug. 2020; Y.J. Lai leg.; SDRI 202108 • 5 gonozooids; South China Sea; 16.092°N, 111.767°E; 8 Aug. 2020; Y.J. Lai leg.; SDRI 202117, 202119, 202121, 202122, 202124 • 1 phorozooid; same data as for preceding; SDRI 202115 • 4 gonozooids; South China Sea; 15.725°N, 112.000°E; 8 Aug. 2020; Y.J. Lai leg.; SDRI 202125, 202126, 202129, 202131 • 1 gonozooid; South China Sea; 17.498°N, 110.661°E; 9 Aug. 2020; Y.J. Lai leg.; SDRI 202101 • 2 gonozooids; South China Sea; 17.114°N, 111.036°E; 9 Aug. 2020; Y.J. Lai leg.; SDRI 202104, 202105 • 2 phorozooids; South China Sea; 18.659°N, 113.798°E; 22 Aug. 2022; Y. Liu leg.; SDRI 202206, 202207 • 1 gonozooid; South China Sea; 17.168°N, 110.798°E; 27 Aug. 2022; Y. Liu leg.; SDRI 202217 • 3 gonozooids; South China Sea; 17.177°N, 114.400°E; 28 Aug. 2022; Y. Liu leg.; SDRI 202220, 202222, 202223 • 1 phorozooid; same data as for preceding; SDRI 202221 • 2 gonozooids; South China Sea; 15.692°N, 114.985°E; 29 Aug. 2022; Y. Liu leg.; SDRI 202229, 202230 • 4 phorozooids; same data as for preceding; SDRI 202224, 202225, 202227, 202228 • 2 gonozooids; South China Sea; 15.656°N, 112.629°E; 30 Aug. 2022; Y. Liu leg.; SDRI 202269, 202270 • 16 gonozooids; South China Sea; 15.666°N, 113.802°E; 30 Aug. 2022; Y. Liu leg.; SDRI 202237, 202240, 202244, 202247, 202248, 202251, 202254, 202255, 202256, 202258, 202259, 202260, 202261, 202262, 202263, 202264 • 7 phorozooids; same data as for preceding; SDRI 202238, 202241, 202242, 202245, 202249, 202250, 202252 • 1 gonozooid; South China Sea; 15.684°N, 111.396°E; 31 Aug. 2022; Y. Liu leg.; SDRI 202279 • 1 phorozooid; same data as for preceding; SDRI 202282.

###### Description.

**Figure 5.**
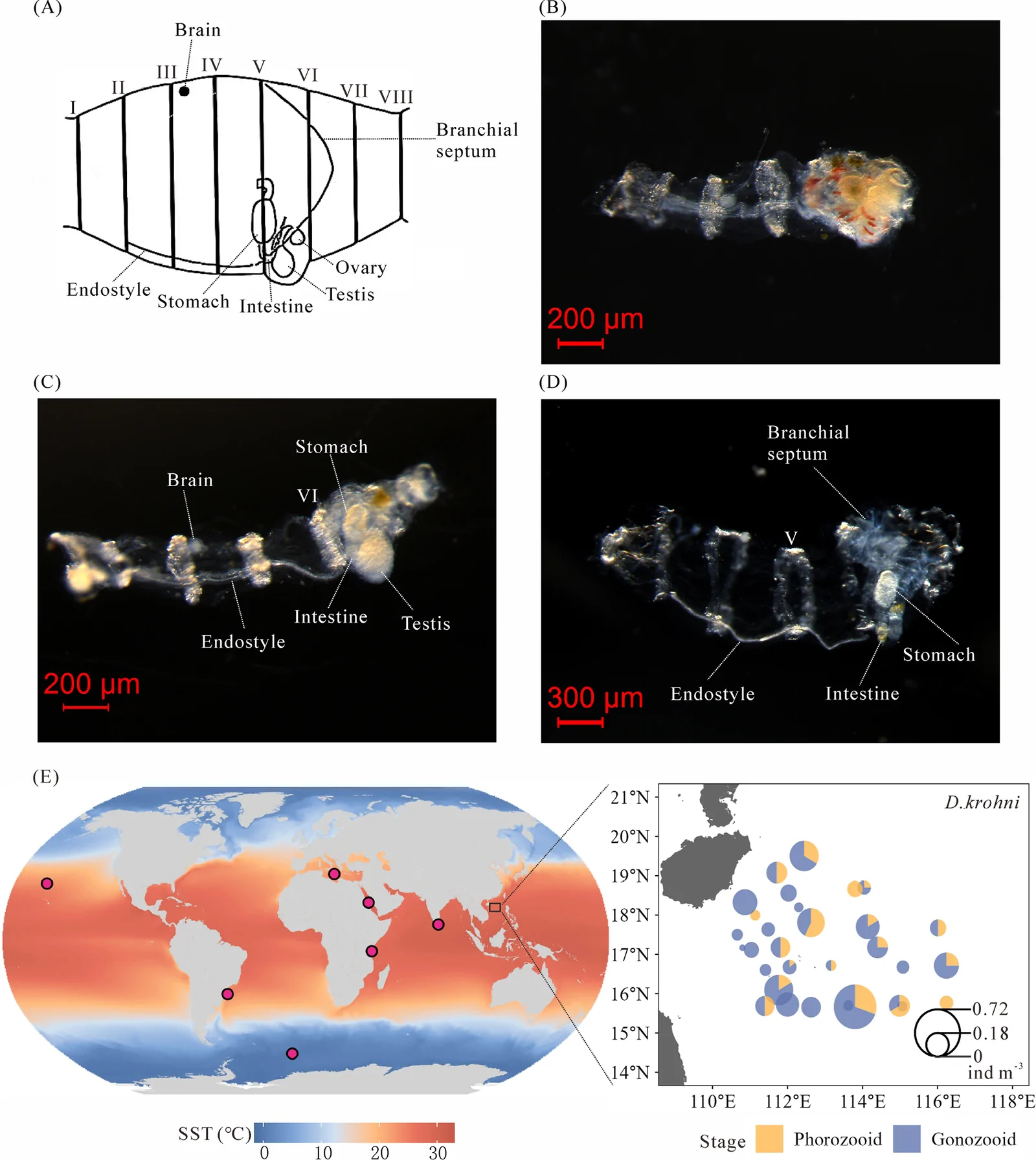
Doliolina (Doliolina) krohni and its distribution. **A**. Schematic diagram of the gonozooid adapted from Godeaux (1998a); **B**. Light micrograph of a gonozooid specimen showing red streaks; **C**. Light micrograph of a phorozooid specimen; **D**. Light micrograph of a gonozooid specimen; **E**. Distribution of *D. krohni* in the South China Sea based on the present study and in the global ocean compiled from historical literature and biodiversity databases, including the GBIF, OBIS, JeDI, SeaLifeBase, and WoRMS. Dots on the global map represent approximate occurrence locations.

Length 0.5–2.4 mm. Long endostyle extending from MII to MV (Fig. 5A–D). Branchial septum arched, between MV and MVI. Brain between MIII and MIV. Testis pear-shaped, swollen, protruding ventrally below intestine. Ovary anterior to MVI. In some specimens, bright red streaks or patches were observed near the stomach, a feature not previously described (Fig. 5B).

###### Remarks.

Most specimens appeared flattened and contracted, likely due to fixation in formaldehyde solution. This species closely resembles Doliolina (Doliolina) müelleri, but can be clearly distinguished by its markedly longer endostyle. Although *D. müelleri* has been reported multiple times in the SCS, *D. krohni* has not been recorded previously. In the present study, however, the pattern was reversed: *D. krohni* occurred frequently, while *D. müelleri* was rarely observed.

###### Distribution.

In this study, this species was widely distributed, exhibiting high occurrence rates in both 2020 (72.73%) and 2022 (63.64%; Table 2). In the southwestern area, phorozooids were the dominant stage and abundances were low. In contrast, higher abundances were observed in the northwestern and southeastern areas, coinciding with the dominance of gonozooid (Fig. 5E). According to the historical literature and biodiversity databases, this species was found in European waters, Mediterranean, Indian Ocean, Arabian Sea (Purushothaman et al. 2018), South Atlantic, Brazilian waters (Da Silva Rodrigues et al. 2024), Red Sea (Godeaux 1987), and the North Pacific (Tokioka 1960).

##### Doliolina (Doliolina) sigmoides

Taxon classificationAnimaliaDoliolidaDoliolidae

Garstang, 1933

A77988D7-7655-56F0-97A8-61B1CF8B1AC8

Fig. 6

###### Material examined.

China • 2 gonozooids; South China Sea; 16.714°N, 116.236°E; 29 Jul. 2020; Y.J. Lai leg.; SDRI 202045, 202047 • 2 phorozooids; same data as for preceding; SDRI 202049 • 1 phorozooid; South China Sea; 17.672°N, 116.014°E; 29 Jul. 2020; Y.J. Lai leg.; SDRI 202040 • 1 gonozooid; South China Sea; 18.686°N, 115.016°E; 22 Aug. 2022; Y. Liu leg.; SDRI 202203 • 1 phorozooid; South China Sea; 17.191°N, 113.196°E; 28 Aug. 2022; Y. Liu leg.; SDRI 202219 • 1 gonozooid; South China Sea; 15.656°N, 112.629°E; 30 Aug. 2022; Y. Liu leg.; SDRI 202267 • 2 phorozooids; same data as for preceding; SDRI 202266, 202268.

###### Description.

**Figure 6.**
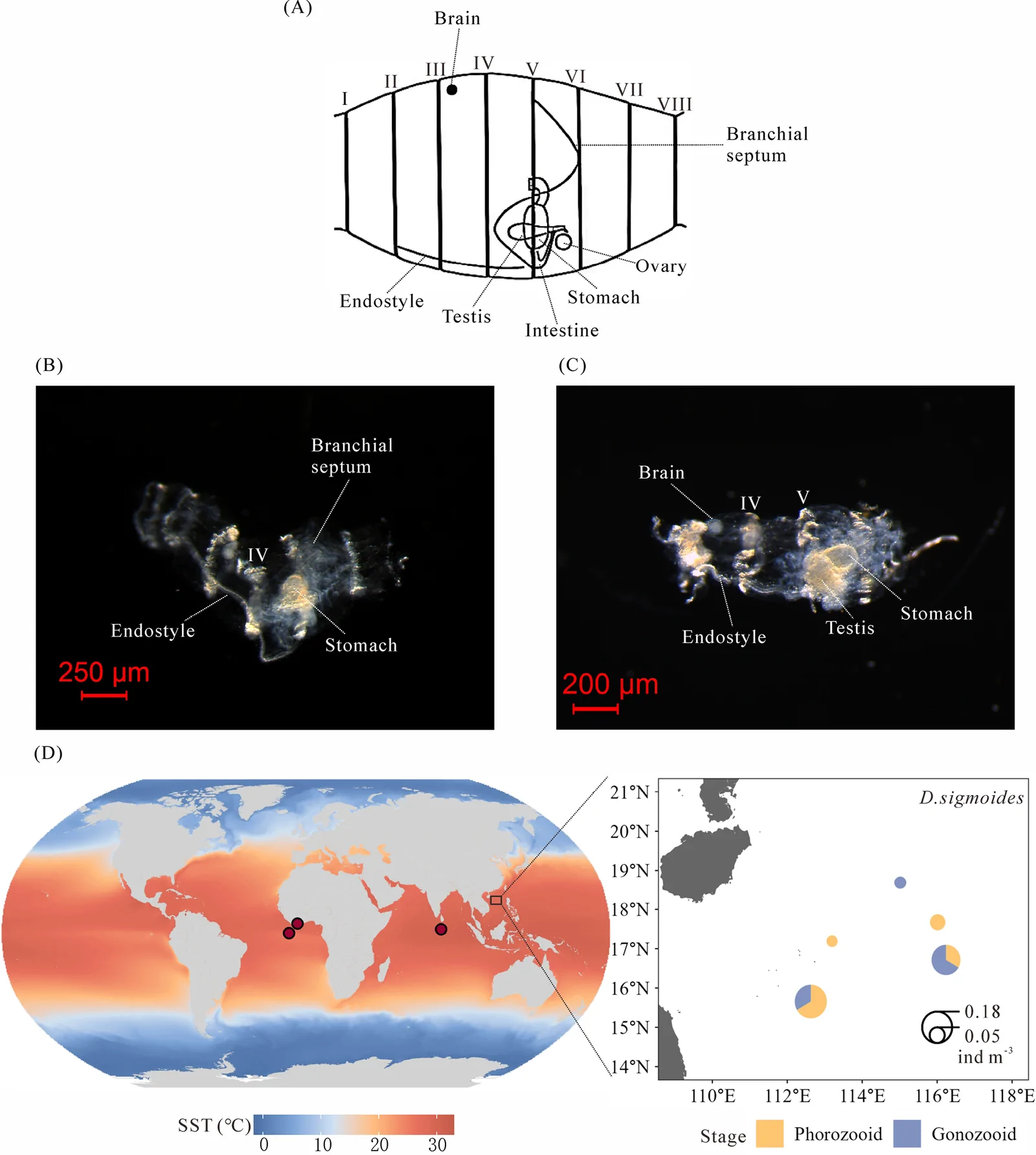
Doliolina (Doliolina) sigmoides and its distribution. **A**. Schematic diagram of the gonozooid adapted from Godeaux (1998a); **B**. Light micrograph of a phorozooid specimen; **C**. Light micrograph of a gonozooid specimen; **D**. Distribution of *D. sigmoides* in the South China Sea based on the present study and in the global ocean compiled from historical literature and biodiversity databases, including the GBIF, OBIS, JeDI, SeaLifeBase, and WoRMS. Dots on the global map represent approximate occurrence locations.

Length 1.0–1.9 mm. Endostyle slender, extending from MII to MV. Branchial septum S-shaped, extending from MV to MVI, folding back to MIV and then returning to MV. Brain located between MIII and MIV. Testis short, horizontal, swollen anterior to MV. Ovary positioned immediately posterior to testis (Fig. 6A–C).

###### Distribution.

This species was recorded in the western part of the study area, with occurrence rates of 6.06% in 2020 and 27.27% in 2022 (Table 2, Fig. 6D). Global records are scarce (Fig. 6D), with reported occurrences primarily limited to the Guinea Basin and the Indian Ocean (Purushothaman et al. 2018).

#### Subgenus: Doliolina (Doliolinetta) Godeaux, 1998

##### Doliolina (Doliolinetta) indica

Taxon classificationAnimaliaDoliolidaDoliolidae

Neumann, 1906

C21B9A61-FE93-5558-9AF7-8739670458B1

Fig. 7

###### Material examined.

China • 1 gonozooid; South China Sea; 17.806°N, 112.625°E; 28 Jul. 2020; Y.J. Lai leg.; SDRI 202016 • 2 gonozooids; South China Sea; 17.703°N, 114.139°E; 28 Jul. 2020; Y.J. Lai leg.; SDRI 202028, 202029 • 2 gonozooids; South China Sea; 17.672°N, 116.014°E; 29 Jul. 2020; Y.J. Lai leg.; SDRI 202037, 202038 • 2 gonozooids; South China Sea; 16.714°N, 116.236°E; 29 Jul. 2020; Y.J. Lai leg.; SDRI 202041, 202046 • 1 phorozooid; South China Sea; 16.675°N, 115.078°E; 29 Jul. 2020; Y.J. Lai leg.; SDRI 202051 • 1 gonozooid; South China Sea; 16.739°N, 114.092°E; 30 Jul. 2020; Y.J. Lai leg.; SDRI 202054 • 1 phorozooid; same data as for preceding; SDRI 202055 • 1 phorozooid; South China Sea; 16.675°N, 112.056°E; 30 Jul. 2020; Y.J. Lai leg.; SDRI 202059 • 2 gonozooids; South China Sea; 17.175°N, 111.814°E; 5 Aug. 2020; Y.J. Lai leg.; SDRI 202075, 202081 • 2 phorozooids; same data as for preceding; SDRI 202079, 202080 • 1 gonozooid; same data as for preceding; SDRI 202078 • 2 gonozooids; South China Sea; 17.114°N, 111.036°E; 9 Aug. 2020; Y.J. Lai leg.; SDRI 202102, 202103 • 2 gonozooids; South China Sea; 16.603°N, 111.414°E; 8 Aug. 2020; Y.J. Lai leg.; SDRI 202106, 202107 • 1 gonozooid; South China Sea; 16.092°N, 111.767°E; 8 Aug. 2020; Y.J. Lai leg.; SDRI 202116 • 3 phorozooids; same data as for preceding; SDRI 202118, 202120, 202123 • 1 gonozooid; South China Sea; 15.725°N, 112.000°E; 8 Aug. 2020; Y.J. Lai leg.; SDRI 202128 • 1 gonozooid; South China Sea; 18.697°N, 114.044°E; 26 Jul. 2020; Y.J. Lai leg.; SDRI 202135 • 1 phorozooid; South China Sea; 15.700°N, 113.625°E; 7 Aug. 2020; Y.J. Lai leg.; SDRI 202140 • 1 gonozooid; same data as for preceding; SDRI 202141 • 2 gonozooids; South China Sea; 15.681°N, 115.058°E; 6 Aug. 2020; Y.J. Lai leg.; SDRI 202149, 202150 • 3 gonozooids; South China Sea; 15.767°N, 116.239°E; 6 Aug. 2020; Y.J. Lai leg.; SDRI 202155, 202157, 202158 • 2 gonozooids; South China Sea; 19.156°N, 112.822°E; 27 Jul. 2020; Y.J. Lai leg.; SDRI 202160, 202161 • 1 gonozooid; South China Sea; 19.503°N, 112.444°E; 27 Jul. 2020; Y.J. Lai leg.; SDRI 202166 • 2 gonozooids; South China Sea; 18.194°N, 112.300°E; 28 Jul. 2020; Y.J. Lai leg.; SDRI 202187, 202189 • 1 gonozooid; South China Sea; 17.168°N, 110.798°E; 27 Aug. 2022; Y. Liu leg.; SDRI 202218 • 1 gonozooid; South China Sea; 15.692°N, 114.985°E; 29 Aug. 2022; Y. Liu leg.; SDRI 202226 • 5 gonozooids; South China Sea; 15.666°N, 113.802°E; 30 Aug. 2022; Y. Liu leg.; SDRI 202246, 202253, 202257 • 1 phorozooid; same data as for preceding; SDRI 202239 • 3 gonozooids; South China Sea; 15.684°N, 111.396°E; 31 Aug. 2022; Y. Liu leg.; SDRI 202278, 202280, 202281.

###### Description.

**Figure 7.**
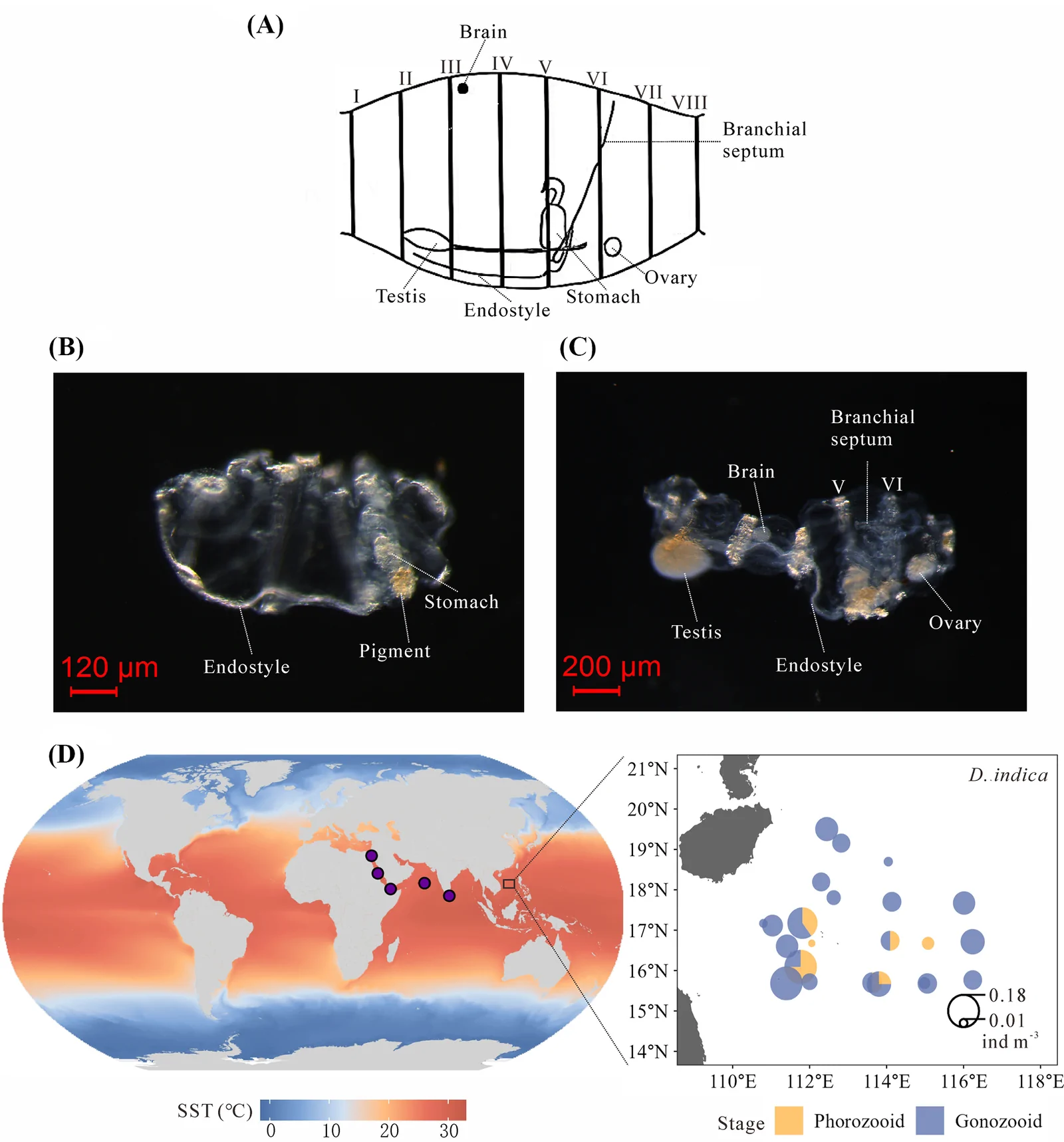
Doliolina (Doliolinetta) indica and its distribution. **A**. Schematic diagram of the gonozooid adapted from Godeaux (1998a); **B**. Light micrograph of a phorozooid specimen; **C**. Light micrograph of a gonozooid specimen; **D**. Distribution of *D. indica* in the South China Sea based on the present study and in the global ocean compiled from historical literature and biodiversity databases, including the GBIF, OBIS, JeDI, SeaLifeBase, and WoRMS. Dots on the global map represent approximate occurrence locations.

Length 0.4–2.1 mm. Endostyle slender, extending from MII to MV. Branchial septum oblique between MV and MVI. Brain located between MIII and MIV. Testis tubular, horizontal, swollen at MII. Stomach masked by a cloud of yellow pigment. Ovary anterior to MVII (Fig. 7A–C).

###### Remarks.

This species is readily distinguishable by the yellow pigment cloud around the stomach both in the phorozooid and gonozooid stages, and by the swollen testis at M II in the gonozooid stage (Fig. 7C).

###### Distribution.

This species was widely distributed within the study area, exhibiting a high occurrence rate of 57.58% in 2020 (Table 2, Fig. 7D). Globally, records are scarce (Fig. 7D) with documented occurrences primarily limited to the Indian Ocean, Gulf of Aden, Red Sea, Gulf of Aqaba, and Arabian Sea (Purushothaman et al. 2018). Its occurrence in the SCS is consistent with its tropical–subtropical distribution and may be facilitated by the SCS acting as a biogeographic transition zone within the Indo–Pacific.

##### Suborder: Doliopsidina

Taxon classificationAnimaliaDoliolidaDoliolidae

Godeaux, 1996

9B8879CC-4D94-5988-B576-6A4B824294D3

Fig. 8

###### Material examined.

China • 1 specimen (sex undefined); South China Sea; 17.806°N, 112.625°E; 28 Jul. 2020; Y.J. Lai leg.; SDRI 202002 • 3 specimens (sex undefined); South China Sea; 17.703°N, 114.139°E; 28 Jul. 2020; Y.J. Lai leg.; SDRI 202025, 202027, 202035 • 1 specimen (sex undefined); South China Sea; 16.675°N, 115.078°E; 29 Jul. 2020; Y.J. Lai leg.; SDRI 202050 • 1 phorozooid; South China Sea; 16.675°N, 112.056°E; 30 Jul. 2020; Y.J. Lai leg.; SDRI 202060 • 7 specimens (sex undefined); same data as for preceding; SDRI 202063, 202064, 202065, 202069, 202070, 202071, 202072 • 5 specimens (sex undefined); South China Sea; 17.175°N, 111.814°E; 5 Aug. 2020; Y.J. Lai leg.; SDRI 202082, 202083, 202084, 202085, 202087 • 2 specimens (sex undefined); South China Sea; 17.994°N, 111.139°E; 4 Aug. 2020; Y.J. Lai leg.; SDRI 202091, 202092 • 3 specimens (sex undefined); South China Sea; 18.325°N, 110.867°E; 4 Aug. 2020; Y.J. Lai leg.; SDRI 202093, 202098, 202099 • 1 specimen (sex undefined); South China Sea; 17.981°N, 110.378°E; 9 Aug. 2020; Y.J. Lai leg.; SDRI 202100 • 6 phorozooids; South China Sea; 16.603°N, 111.414°E; 8 Aug. 2020; Y.J. Lai leg.; SDRI 202109, 202110, 202111, 202112, 202113, 202114 • 1 phorozooid; South China Sea; 15.725°N, 112.000°E; 8 Aug. 2020; Y.J. Lai leg.; SDRI 202127 • 2 specimens (sex undefined); same data as for preceding; SDRI 202130, 202132 • 1 phorozooid; South China Sea; 18.697°N, 114.044°E; 26 Jul. 2020; Y.J. Lai leg.; SDRI 202133 • 7 specimens (sex undefined); South China Sea; 15.681°N, 115.058°E; 6 Aug. 2020; Y.J. Lai leg.; SDRI 202143, 202144, 202145, 202146, 202147, 202148, 202151 • 3 specimens (sex undefined); South China Sea; 15.767°N, 116.239°E; 6 Aug. 2020; Y.J. Lai leg.; SDRI 202152, 202154, 202156 • 7 specimens (sex undefined); South China Sea; 19.503°N, 112.444°E; 27 Jul. 2020; Y.J. Lai leg.; SDRI 202162, 202164, 202165, 202168, 202170, 202171, 202172 • 4 specimens (sex undefined); South China Sea; 18.558°N, 112.028°E; 27 Jul. 2020; Y.J. Lai leg.; SDRI 202178, 202179, 202180, 202181 • 1 specimen (sex undefined); South China Sea; 18.194°N, 112.300°E; 28 Jul. 2020; Y.J. Lai leg.; SDRI 202183 • 1 phorozooid; same data as for preceding; SDRI 202184 • 6 specimens (sex undefined); same data as for preceding; SDRI 202185, 202186, 190, 192, 193, 194, 195 • 1 phorozooid; same data as for preceding; SDRI 202188 • 4 specimens (sex undefined); South China Sea; 18.686°N, 115.016°E; 22 Aug. 2022; Y. Liu leg.; SDRI 202201, 202202, 202204, 202205 • 6 specimens (sex undefined); South China Sea; 18.659°N, 113.798°E; 22 Aug. 2022; Y. Liu leg.; SDRI 202208, 202209, 202210, 202211, 202212, 202213 • 3 specimens (sex undefined); South China Sea; 18.674°N, 112.594°E; 23 Aug. 2022; Y. Liu leg.; SDRI 202214, 202215, 202216 • 6 specimens (sex undefined); South China Sea; 15.692°N, 114.985°E; 29 Aug. 2022; Y. Liu leg.; SDRI 202231, 202232, 202233, 202234, 202235, 202236 • 2 specimens (sex undefined); South China Sea; 15.666°N, 113.802°E; 30 Aug. 2022; Y. Liu leg.; SDRI 202243, 202265 • 7 specimens (sex undefined); South China Sea; 15.656°N, 112.629°E; 30 Aug. 2022; Y. Liu leg.; SDRI 202271, 202272, 202273, 202274, 202275, 202276, 202277.

###### Description.

**Figure 8.**
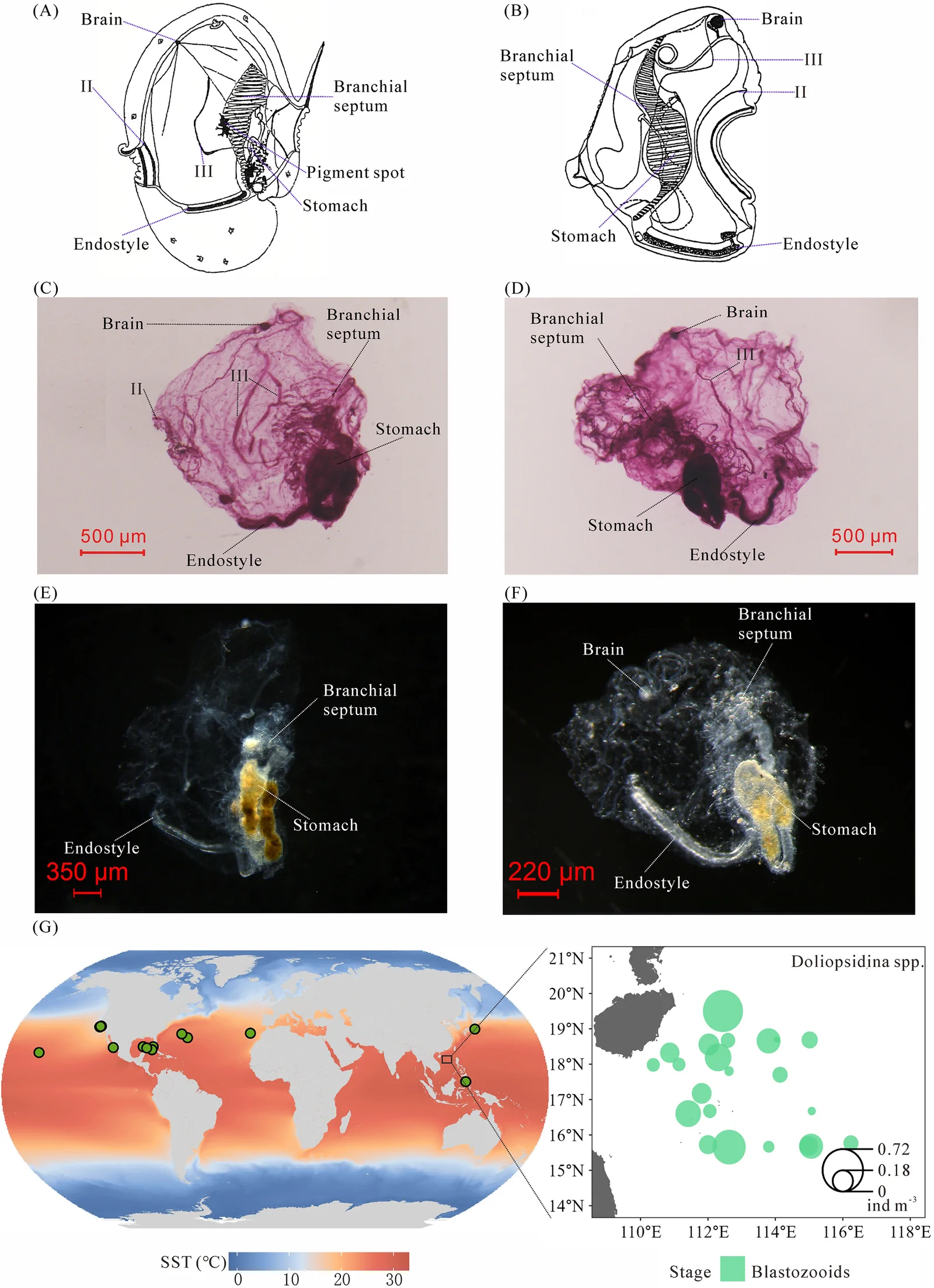
Doliopsidina spp. and their distribution. **A**. Schematic diagram of the gonozooid of *Doliopsis rubescens* adapted from Kowalevsky and Barrois (1883); **B**. Schematic diagram of *Paradoliopsis harbisoni* adapted from Godeaux (2003); **C**. Light micrograph of *Doliopsis* sp. after staining with Rose Bengal sodium salt; **D**. Light micrograph of *Paradoliopsis* sp. after staining with Rose Bengal sodium salt; **E**. Light micrograph of an undefined specimen; **F**. Light micrograph of another undefined specimen; **G**. Distribution of Doliopsidina spp. in the South China Sea based on the present study and in the global ocean compiled from historical literature and biodiversity databases, including the GBIF, OBIS, JeDI, SeaLifeBase, and WoRMS. Dots on the global map represent approximate occurrence locations.

Length 0.7–6.4 mm. The suborder Doliopsidina is distinguished from the more commonly reported suborder Doliolidina by the presence of only five muscle bands, in contrast to the eight muscle bands characteristic of the latter (Godeaux 2003; Robison et al. 2005). The five muscle bands are not arranged in parallel. In the specimens examined in this study, the muscle bands were slender and transparent, and the digestive tract was U-shaped (Fig. 8C–F).

###### Remarks.

Owing to the extreme slenderness and transparency of the muscle bands, most individuals of this suborder could not be identified to species or even family level. After staining, some specimens could be assigned to *Doliopsis* sp. (Fig. 8C) and *Paradoliopsis* sp. (Fig. 8D), whereas the morphological characters observed in other specimens indicate the presence of additional, unresolved taxa within Doliopsidina.

###### Distribution.

The suborder Doliopsidina comprises three families, four genera, and five described species, none of which have previously been reported from the SCS. All known members of Doliopsidina are warm-temperate species. Based on the global records (Fig. 8G), Doliopsidina species have been recorded from the coastal waters off Japan, the Pacific coast of the United States, the California Current system, the North Atlantic, the Caribbean Sea, the Mediterranean Sea, and the Gulf of Mexico.

## Discussion

This study provides the most comprehensive taxonomic assessment of Thaliacea conducted to date in the SCS, documenting 28 species, which represents the highest richness reported for this region (Table 1). Previous studies conducted in adjacent areas reported lower richness, with only 18 and 23 species recorded (Lin and Lin 2006; Li et al. 2016). Methodological differences may partly account for this discrepancy, as earlier surveys employed 500 μm nets, whereas the finer mesh used in the present study (330 μm) is more effective at retaining small-sized thaliaceans, particularly doliolids. Differences in net type and mesh size are well known to influence observed zooplankton assemblages (Garcia et al. 2021).

However, methodological differences alone cannot explain the exceptionally high richness observed, as studies conducted elsewhere in the SCS using comparable mesh sizes have not reported similarly high thaliacean diversity (Table 1). Moreover, the species richness documented here is also unusually high compared with that of other regions where thaliacean diversity and ecology have been relatively well studied, including the North Atlantic, the Pacific Ocean, and various coastal systems (Van Soest 1975; Martin et al. 2016; Ishak et al. 2020, 2022; Pitt et al. 2023; Da Silva Rodrigues et al. 2024). The exceptionally high species richness observed in the SCS likely reflects the combined influence of its biogeographic setting and local physical forcing. During the sampling period, monsoon-driven circulation, mesoscale cyclonic and anticyclonic eddies, and coastal upwelling generated pronounced hydrographic heterogeneity (Suppl. material 1), which likely enhanced habitat diversity and facilitated the coexistence of species with different ecological affinities (Liu 2013; Diao et al. 2022). This physical complexity may partly account for the higher thaliacean richness observed relative to other regions of the SCS. Moreover, as a central component of the Indo–Pacific biogeographic province, the SCS may function as a biological convergence zone, allowing the co-occurrence of thaliacean species from the tropical Indian Ocean, the western Pacific, and locally adapted taxa (Diao et al. 2022; Liu et al. 2024; Lai et al. 2025), which further contributes to the elevated species richness compared with other regions globally.

However, the lack of time-series data, multi-gear sampling, and standardized regional comparisons constrain our interpretation of this high diversity. It remains unclear whether this pattern represents a localized and seasonal phenomenon or reflects actual shifts in thaliacean species composition, possibly driven by global climate change. These findings underscore the need for long-term, multi-method monitoring of thaliaceans.

This study documents five newly recorded species and one suborder of Doliolidae, thereby expanding current knowledge of doliolid diversity and distribution in the SCS. The discovery of *D. mirabilis* and *D. tritonis* fills a taxonomic gap for the genus *Dolioletta* in the SCS, which was previously only represented by *D. gegenbauri*. Both species have been rarely reported globally, likely due to their morphological similarity to *D. gegenbauri*, particularly during the phorozooid stage (Godeaux 1998a), which may have led to frequent misidentification.

Three other new species belong to the genus *Doliolina*. This genus has been rarely documented in previous studies of the SCS but was frequently encountered in this study (Table 2). *D. indica* has previously been reported mainly from the Indian Ocean, Arabian Sea, and Red Sea (Fig. 7D). Its occurrence in the SCS is consistent with its tropical-subtropical distribution and may be facilitated by the SCS acting as a biogeographic transition zone within the Indo-Pacific. *D. krohni* is considered one of the few doliolid species capable of thriving under both tropical and subpolar conditions (Weikert and Godeaux 2008). Although suitable niches likely exist in the SCS, this species is recorded here for the first time. *D. sigmoides* was rarely recorded in previous studies. In this study, it occurred more frequently in offshore waters (Fig. 6D), which may account for its rare records in conventional coastal surveys.

The suborder Doliopsidina, although first described in 1996, had not previously been recorded in the SCS. Its detection in this study therefore filled a major biogeographic gap. In this study, Doliopsidina exhibited relatively high occurrence rates, reaching up to 54.55%. All known members of Doliopsidina are warm-temperate species, and thus, the occurrence of Doliopsidina in the SCS is biogeographically plausible. However, determining whether the observed specimens represent any known species or a potentially undescribed taxon will require additional material and further investigation.

Overall, these findings suggest that doliolids in the SCS are far more diverse and ecologically differentiated than previously recognized, with important implications for regional biodiversity assessments and for the reevaluation of the biogeographic patterns of pelagic tunicates.

## Conclusions

This study provides the most comprehensive assessment of thaliaceans in the SCS to date, documenting 28 thaliacean species, the highest species richness reported in this region. These include five newly recorded species and the first record of the suborder Doliopsidina in the SCS. These findings reveal an unusually complex species composition and ecological differentiation, highlighting the previously underestimated diversity of thaliaceans in the SCS.

Despite increasing global trends of biodiversity loss under climate warming and ocean acidification, we observed exceptionally high thaliacean diversity using plankton net trawling. This suggests that marine biodiversity may still be substantially underestimated and that our understanding of pelagic ecosystem diversity and function requires further refinement.

However, as this study was conducted during a single season, we cannot determine whether the observed high diversity is a localized, seasonal phenomenon or reflects actual shifts in thaliacean species composition potentially linked to global climate change. These uncertainties highlight the need for establishing long-term, cross-regional monitoring networks that integrate multiple sampling approaches, including eDNA and in situ imaging technologies, to achieve a more comprehensive understanding of gelatinous zooplankton diversity and ecosystem function.

## Supplementary Material

XML Treatment for Dolioletta mirabilis

XML Treatment for Dolioletta tritonis

XML Treatment for Doliolina (Doliolina) krohni

XML Treatment for Doliolina (Doliolina) sigmoides

XML Treatment for Doliolina (Doliolinetta) indica

XML Treatment for Doliopsidina

## Additional information

### Artificial Intelligence (AI) use

The authors accept full responsibility for the content of the manuscript, including the disclosure of any use of AI.

No AI tools were used in the preparation of this manuscript.

### Author ORCIDs

Y. Lai https://orcid.org/0000-0002-1936-6067

Z. Zheng https://orcid.org/0009-0006-0144-1715

M. Chen https://orcid.org/0009-0005-7950-5953

Y. Tan https://orcid.org/0000-0002-6378-2156

### Data availability

All of the data that support the findings of this study are available in the main text or Supplementary Information.

## Supplementary materials

Supplementary material 1 Distribution data of environmental factors Data type docx Explanation note This supplementary file offer the distribution data of environmental factors during sampling period. https://binary.pensoft.net/file/1751757This dataset is made available under the Open Database License (http://opendatacommons.org/licenses/odbl/1.0/). The Open Database License (ODbL) is a license agreement intended to allow users to freely share, modify, and use this Dataset while maintaining this same freedom for others, provided that the original source and author(s) are credited. Yanjiao Lai, Zeqi Zheng, Mianrun Chen, Yehui Tan
