# Supplementary material for: New species and suborder records reveal unusually high thaliacean (Chordata, Tunicata) diversity in the South China Sea
Source: Zookeys. 2026 Aug 21;1290:147–70. doi: 10.3897/zookeys.1290.188516 (PMC13525402; doi:10.3897/zookeys.1290.188516)
Supplement: Supplementary file 1 [file zookeys-1290-147_article-188516__-s001.docx]

**Environmental factor**

During the summer of 2020, satellite-derived salinity maps indicated the presence of a low-salinity water mass originating from the Mekong River in the southwestern part of the study area from one week prior to sampling until the end of the sampling period (Fig. 1 a–c). Sea level anomaly (SLA) maps revealed a warm eddy extending from the northeast to the southwest across the study region (Fig. 1 d). The sea surface temperature (SST) ranged from 29.97 to 31.65 °C, generally showing lower temperatures in the west and higher temperatures in the east. Under the influence of the Qiongdong upwelling, a persistent cold water mass near the western coast (station S24) extended eastward to the central station S11. In contrast, relatively high SSTs (mostly above 31 °C) were observed at most central and eastern stations, consistent with the center of the warm eddy (Fig. 1 e). Surface salinity varied between 33.72 and 34.23, with higher values in the north and lower values in the south. Notably, salinity in the southwestern area was generally below 34, which is consistent with the low-salinity plume from the Mekong River shown in the satellite salinity images (Fig. 1 f). Chlorophyll-a concentration ranged from 0.03 to 0.40 μg L⁻¹, exhibiting a spatial distribution pattern corresponding to that of SST. Higher chlorophyll-a concentrations (mostly >0.1 μg L⁻¹) were found in the western upwelling-affected area, with the maximum recorded at station S9, whereas lower values were observed in the eastern region influenced by the warm eddy (Fig. 1 g).

**Figure 1.** Remote sensing salinity before and during the sampling period (a‒c). Sea level anomaly during the sampling period (d). Sea surface temperature (e), sea surface salinity (f), and sea surface chlorophyll *a* (g) obtained from the conductivity, temperature, and depth instrument during the sampling period. Numbers on the map identify the sampling stations.

In 2022, according to the SLA map (Fig. 2d), the southernmost transect stations were located at the edge of a warm eddy. Sea surface temperature (SST) ranged from 29.07 to 30.33 °C, with higher values observed in the western region and lower values in the central and southern regions (Fig. 2a). SSS varied between 33.19 and 33.52, characterized by lower salinity in the southern area, where a low-salinity water mass extended from the central region toward the north (Fig. 2b). Surface chlorophyll-a concentration ranged from 0.07 to 0.10 μg L⁻¹ and exhibited a decreasing trend from west to east (Fig. 2c).

**Figure 2.** Sea surface temperature (a), sea surface salinity (b), and sea surface chlorophyll *a* (c) obtained from the conductivity, temperature, and depth instrument during the sampling period. Sea level anomaly during the sampling period (d).
